# Elimination of a Free Cysteine by Creation of a Disulfide Bond Increases the Activity and Stability of Candida boidinii Formate Dehydrogenase

**DOI:** 10.1128/AEM.02624-16

**Published:** 2016-12-30

**Authors:** Junxian Zheng, Taowei Yang, Junping Zhou, Meijuan Xu, Xian Zhang, Zhiming Rao

**Affiliations:** The Key Laboratory of Industrial Biotechnology, Ministry of Education, Jiangnan University, Wuxi, China; Michigan State University

**Keywords:** Candida boidinii, cysteine, disulfide bond, formate dehydrogenase, site-directed mutagenesis

## Abstract

NAD^+^-dependent formate dehydrogenase (FDH; EC 1.2.1.2) is an industrial enzyme widely used for NADH regeneration. However, enzyme inactivation caused by the oxidation of cysteine residues is a flaw of native FDH. In this study, we relieved the oxidation of the free cysteine of FDH from Candida boidinii (*Cbo*FDH) through the construction of disulfide bonds between A10 and C23 as well as I239 and C262. Variants A10C, I239C, and A10C/I239C were obtained by the site-directed mutagenesis and their properties were studied. Results showed that there were no significant changes in the optimum temperature and pH between variants and wild-type *Cbo*FDH. However, the stabilities of all variant enzymes were improved. Specifically, the *Cbo*FDH variant A10C (A10C_*fdh*_) showed a significant increase in copper ion resistance and acid resistance, a 6.7-fold increase in half-life at 60°C, and a 1.4-fold increase in catalytic efficiency compared with the wild type. Asymmetric synthesis of l-*tert*-leucine indicated that the process time was reduced by 40% with variant A10C_*fdh*_, which benefited from the increase in catalytic efficiency. Circular dichroism analysis and molecular dynamics simulation indicated that variants that contained disulfide bonds lowered the overall root mean square deviation (RMSD) and consequently increased the protein rigidity without affecting the secondary structure of enzyme. This work is expected to provide a viable strategy to avoid the microbial enzyme inactivation caused by the oxidation of the free cysteine residues and improving their performances.

**IMPORTANCE** FDH is widely used for NADH regeneration in dehydrogenase-based synthesis of optically active compounds to decrease the cost of production. This study highlighted a viable strategy that was used to eliminate the oxidation of free cysteine residues of FDH from Candida boidinii by the introduction of disulfide bonds. Using this strategy, we obtained a variant FDH with improved activity and stability. The improvement of activity and stability of FDH is expected to reduce its price and then further to decrease the cost of its application.

## INTRODUCTION

NAD^+^-dependent formate dehydrogenase (FDH; EC 1.2.1.2) is a homodimeric enzyme which does not contain metal ions or prosthetic groups and catalyzes the oxidation of formate ion to carbon dioxide, coupled with reduction of NAD^+^ to NADH (1). FDHs are widely coupled with other dehydrogenases for the synthesis of chiral intermediates and fine chemicals (2).

The oxidation of cysteine residues with oxygen or their modification with microimpurities (such as Cu^2+^) in the solution are major reasons for the inactivation of most native FDHs (1, 3). Although site-directed mutagenesis of cysteine residues can avoid FDH inactivation caused by the oxidation of the free sulfhydryl groups of cysteine residues, the thermal stability of FDHs is reduced. For example, the rate of thermal inactivation of the variants C23S, C262V, C23S/C262A, and C23S/C262V of FDH from Candida boidinii (*Cbo*FDH) increases drastically (4), and the thermal stability of FDH from Pseudomonas sp. strain 101 also decreases significantly after the replacement of C255 (5). Cysteine residues are frequently found within functional (regulatory, catalytic, or binding) sites in proteins and play an important role in impartation of specialized properties for key sites (6, 7). Thus, the replacement of the free cysteine residues of FDHs is not the best strategy to improve enzyme chemical stability. As a feasible strategy, the construction of disulfide bonds is widely applied in improving the thermal stability of enzymes such as α-amylase (8), xylanases (9), alkaline protease (10), and lipases (11). There are no reports that disulfide bonds can be formed in wild-type FDHs. New disulfide bonds are introduced in FDH from Candida methylica (*Cm*FDH), but the variants are less active and less thermostable than wild-type *Cm*FDH (12). Instead, a strategy of methionine-to-cysteine substitution has succeeded in increasing the thermal stability of *Cm*FDH (13). In addition, other methods, like directed evolution (7) or chemical modification (14), also have succeeded to improve the stability of FDH.

In this study, our goal was to eliminate the oxidation of the free cysteine residues and improve the stability without affecting other biochemical properties of *Cbo*FDH through the rational design of disulfide bonds. The alanine residue at position 10 was mutated to cysteine to form a disulfide bond with C23, and the isoleucine residue at position 239 was mutated to cysteine to form a disulfide bond with C262. The *Cbo*FDH variants A10C, I239C, and A10C/I239C were obtained to characterize biochemical properties, such as thermal stability, pH stability, and catalytic efficiency, and compare them with those of wild-type *Cbo*FDH. We found that performances of FDH A10C variant A10C_*fdh*_ were improved, and the increase in catalytic efficiency could reduce the processing time of l-*tert*-leucine synthesis. Hence, we explored possible mechanisms causing differences in biochemical properties between *Cbo*FDH and its variants by analyzing the model structures and molecular dynamics. The strategy described here may be useful for relieving other cases of microbial enzyme inactivation caused by the oxidation of free cysteines and improving their performance.

## RESULTS

### Selection of target residues for introducing disulfide bonds.

To stabilize the unpaired cysteine residues of *Cbo*FDH against oxidation, two disulfide bonds were designed in C23 and C262, respectively. A PDB file (entry 5DN9) of the three-dimensional (3D) structure model of *Cbo*FDH was submitted to Disulfide by Design (http://cptweb.cpt.wayne.edu/DbD2/index.php) (15). Residue A10 was selected to pair with C23, and residue I239 was selected to pair with C262 according to the size of dihedral angles, χ_3_ (16). The mutants A10C, I239C, and A10C/I239C were constructed, and the derivatives of *Cbo*FDH were denoted A10C_*fdh*_, I239C_*fdh*_, and A10C/I239C_*fdh*_.

### Expression and purification of enzymes.

Residues A10 and I239 were replaced by cysteine for introducing a disulfide bond through site-directed mutagenesis, and the mutant genes (*fdh-A10C*, *fdh-I239C*, and *fdh-A10C/I239C*) were verified by DNA sequencing. They were inserted into plasmid pET-28a(+), and then the recombination plasmids were expressed in Escherichia coli BL21(DE3) to obtain derivatives A10C_*fdh*_, I239C_*fdh*_, and A10C/I239C_*fdh*_. After purification through a nickel affinity chromatography column, the purified enzymes were used for study of enzyme activities and properties.

### Verification of disulfide bond formation.

To determine whether disulfide bonds were introduced into variant enzymes, the purified enzymes (under reducing and nonreducing conditions) were analyzed by 12% SDS-PAGE (shown in Fig. S2 in the supplemental material). The samples of nonreduced enzymes (without dithiothreitol [DTT]) showed slightly faster mobility than reduced enzymes (treated by DTT), except for the wild-type enzyme. Since the reducing reagent DTT can disrupt disulfide bonds in protein, the mobility was changed between nonreduced and reduced variant enzymes. The result suggested that additional disulfide bonds were formed in the variants.

To further verify that disulfide bonds were formed, we detected the amount of free cysteines per milligram of protein and calculated the number of free cysteines in a molecule, including wild-type *Cbo*FDH and its variants. The total number of disulfide bonds in one protein molecule was deduced by subtracting the total number of free cysteines from that of cysteines. The total number of cysteines in wild-type and variant enzymes can be obtained from the amino acid sequence. As expected, the result showed that one disulfide bond was formed in variants A10C_*fdh*_ and I239C_*fdh*_, two disulfide bonds were formed in A10C/I239C_*fdh*_, and no disulfide bond was present in wild-type enzyme *Cbo*FDH (Table 1).

**TABLE 1:** Total numbers of disulfide bonds in wild-type and variant enzymes

Enzyme	Concn of free cysteine (μmol mg^−1^ protein)	Calculated total no. of free cysteines (per protein molecule)	Total no. of cysteines (per protein molecule)	Deduced total no. of disulfide bonds (per protein molecule)
*Cbo*FDH	0.047 ± 0.03	2	2	0
A10C*_fdh_*	0.021 ± 0.02	1	3	1
I239C*_fdh_*	0.024 ± 0.05	1	3	1
A10C/I239C*_fdh_*	0.000	0	4	2

### Copper ion resistance assays.

**FIG 1:**
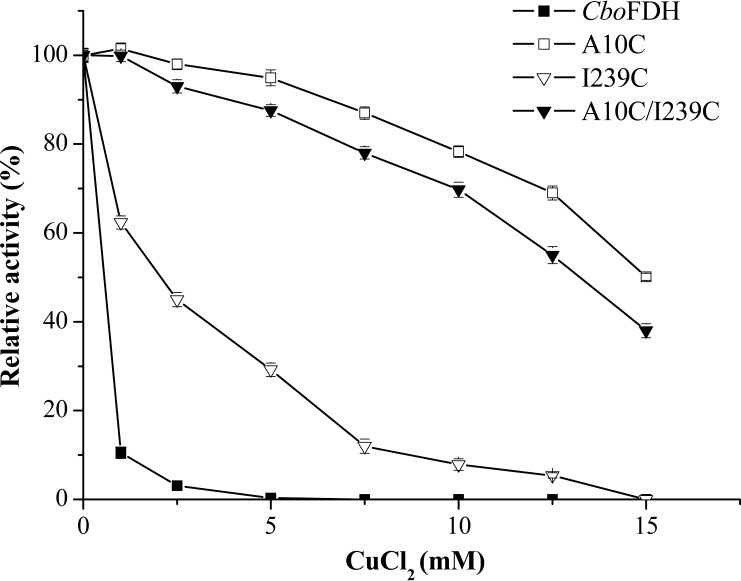
Enzyme assays of wild-type *Cbo*FDH and its mutants in the presence of different concentrations of copper chloride. All of the experiments were performed in triplicate. Standard deviations of the biological replicates are represented by error bars, and the error bars of some data are too small to be seen.

Divalent copper ion (Cu^2+^) is recognized as a transition metal ion that promotes oxidation of sulfhydryl groups (17, 18). In order to further verify the correct formation of disulfide bonds, the activities of wild-type *Cbo*FDH and its derivatives were determined in Tris-HCl buffer (100 mM, pH 7.0) containing different concentrations of Cu^2+^. The results (Fig. 1) showed that the tolerance to Cu^2+^ of all variant enzymes was significantly improved. In particular, variants A10C_*fdh*_ and A10C/I239C_*fdh*_ retained 50.1% and 37.9% activity, respectively, in the presence of 15 mM Cu^2+^, whereas wild-type *Cbo*FDH was completely inactivated by 5 mM Cu^2+^. The improvement of the tolerance to Cu^2+^ of variants to some extent indicated that the formation of disulfide bonds removed the oxidation of sulfhydryl groups.

### Kinetic parameters and specific activity.

The enzyme activity and Michaelis-Menten type of kinetics were measured at 30°C and pH 7.0 in 100 mM potassium phosphate buffer. As shown in Table 2, compared with the specific activity of wild-type enzyme *Cbo*FDH, the specific activities of A10C_*fdh*_ were improved and of I239C_*fdh*_ were declined. The *K_m_* values (for NAD^+^) of A10C_*fdh*_, I239C_*fdh*_, and A10C/I239C_*fdh*_ were higher than that of *Cbo*FDH, indicating that NAD^+^ affinity was slightly reduced. The *K_m_* value (for formate) of I239C_*fdh*_ was lower than that of *Cbo*FDH. This result showed that formate affinity of I239C_*fdh*_ was increased. The (*k*_cat_/*K_m_*)^NAD+^ values showed that the catalytic efficiencies (for NAD^+^) of I239C_*fdh*_ and A10C/I239C_*fdh*_ were decreased. Since the *k*_cat_ value of A10C_*fdh*_ was remarkably improved, the catalytic efficiency (NAD^+^) of A10C_*fdh*_ was increased.

**TABLE 2:** Enzyme kinetic parameters of *Cbo*FDH and the variant enzymes

Enzyme	*K_m_* (*P* value^*a*^)		*k*_cat_ (s^−1^) (*P* value)	(*k*_cat_/*K_m_*)^NAD+^ (μM^−1^ s^−1^) (*P* value)	Sp act (U mg^−1^) (*P* value)
	NAD^+^ (μM)	Formate (mM)			
*Cbo*FDH	53.6 ± 3.4	7.3 ± 0.6	3.3 ± 0.3	0.062 ± 0.0067	5.6 ± 0.4
A10C*_fdh_*	74.2 ± 2.6 (0.001)	8.2 ± 0.4 (0.097)	6.2 ± 0.5 (0.001)	0.084 ± 0.0027 (0.006)	7.4 ± 0.5 (0.008)
I239C*_fdh_*	132.2 ± 4.3 (<0.001)	5.2 ± 0.5 (0.010)	2.6 ± 0.4 (0.072)	0.020 ± 0.0020 (<0.001)	4.2 ± 0.2 (0.006)
A10C/I239C*_fdh_*	119.1 ± 3.8 (<0.001)	5.8 ± 0.8 (0.060)	4.3 ± 0.5 (0.041)	0.036 ± 0.0023 (0.003)	6.3 ± 0.4 (0.099)

a *P* values were used to determine the statistical significance of the differences in kinetic values between variants and the wild type.

### Thermal and pH stability.

**FIG 2:**
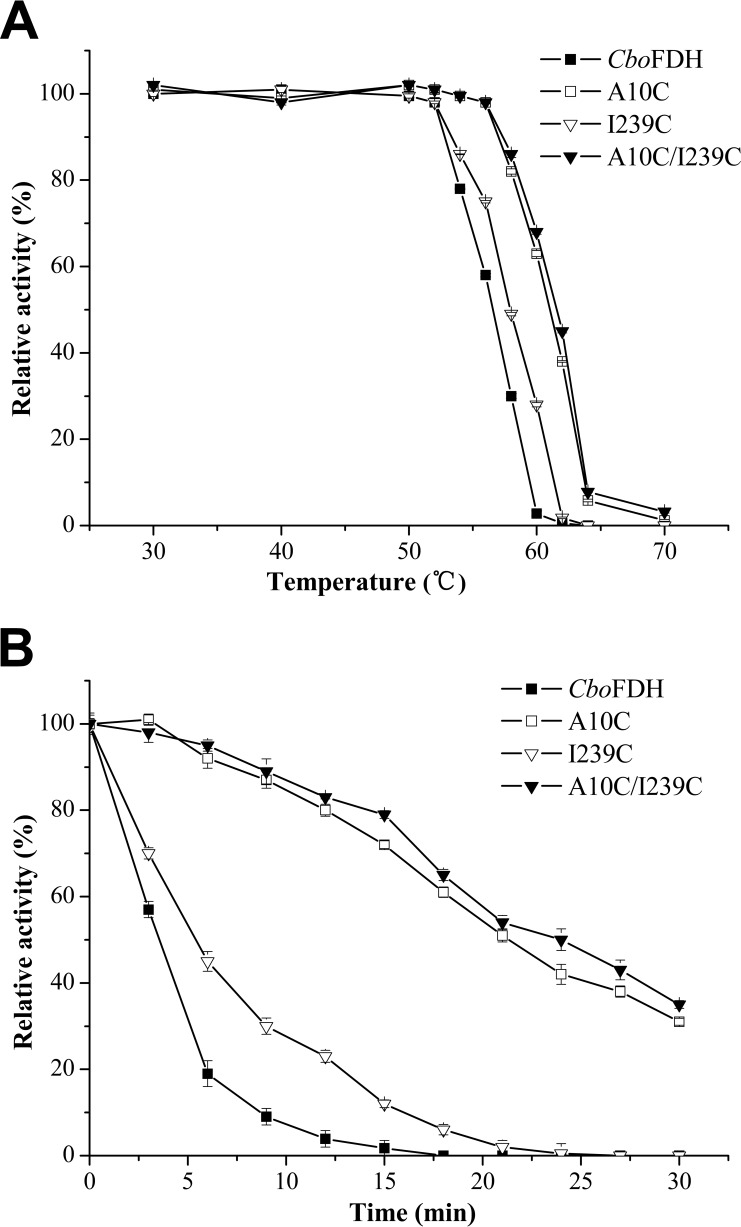
Enzyme stability of wild-type *Cbo*FDH and its mutants. (A) Enzyme inactivation assay at different temperatures for 20 min; (B) time courses of thermal inactivation at 60°C. All of the experiments were performed in triplicate. Standard deviations of the biological replicates are represented by error bars, and the error bars of some data are too small to be seen.

There were no significant differences between wild-type *Cbo*FDH and its variants for the wide pH optimum (pH 6.5 to 9.5) and the broad temperature optimum (45 to 55°C) (data were not shown). The wild-type *Cbo*FDH and its variants were incubated in Tris-HCl (50 mM pH 7.5) buffer for 20 min at a range of temperatures from 30°C to 70°C, and the activities of these enzymes decreased sharply within a short time in a narrow temperature range (Fig. 2A). The *T*_50_ (temperature at which 50% of the initial enzyme activity was lost after heat treatment) values of A10C_*fdh*_ (61.2°C), I239C_*fdh*_ (57.1°C), and A10C/I239C_*fdh*_ (61.8°C) were only slightly higher than that of wild-type *Cbo*FDH (56.7°C), and over 95% of activity was retained after incubation in Tris-HCl (50 mM, pH 7.0) buffer at 50°C for 540 min in the case of wild-type FDH and variants. Hence, in order to further distinguish the differences in thermal stabilities, these enzymes were incubated at 60°C for different times to detect the half-lives of thermal inactivation (*t*_1/2_). As shown in Fig. 2B, variants A10C_*fdh*_ and A10C/I239C_*fdh*_ were significantly more stable than wild-type *Cbo*FDH and variant I239C_*fdh*_. The inactivation process followed first-order kinetics (shown in Fig. S1), and the *t*_1/2_ values at 60°C of A10C_*fdh*_ (21.6 min), I239C_*fdh*_ (5.1 min), and A10C/I239C_*fdh*_ (24.8 min) increased by 6.7, 1.6, and 7.8 times, respectively, compared to that of wild-type *Cbo*FDH (3.2 min). However, the *t*_1/2_ values of variants were similar to that of the wild-type enzyme under reducing conditions (the disulfide bond was broken in the presence of a high concentration of DTT) (data not shown). These results showed that disulfide bonds played a key role in the improvement of variant enzyme stability.

To study the pH stability of variant enzymes, all enzymes were stored for 1 h at 37°C, pH 3.0 to 12.0, and then their residual activities were assayed at 30°C and pH 7.0 in 100 mM potassium phosphate buffer (Fig. S3). The stabilities of the wild-type and variant enzymes were essentially quite similar at pH 5.0 to 12.0, and it was apparent that A10C_*fdh*_ retained higher activity than wild-type *Cbo*FDH and other variant enzymes under extremely acidic (pH 3.0 and 4.0) conditions.

### Asymmetric synthesis with coenzyme regeneration.

**FIG 3:**
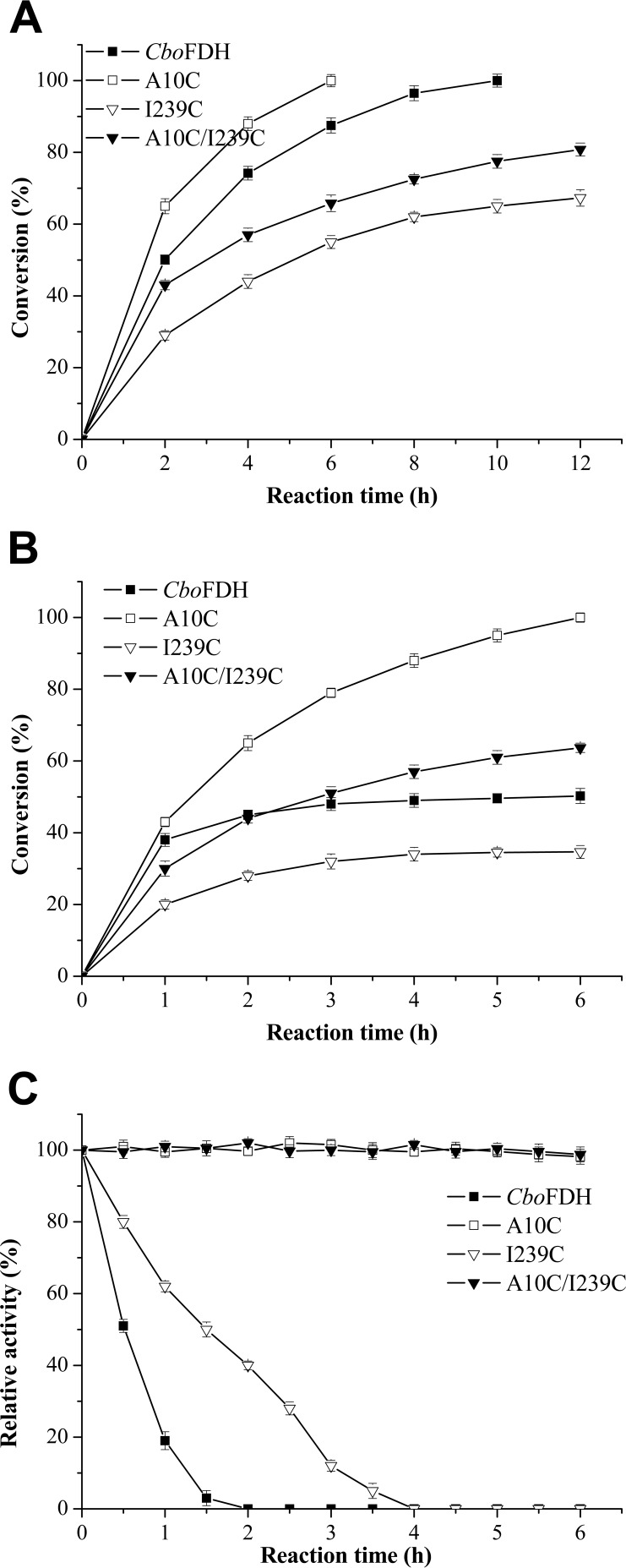
Asymmetric synthesis of l-*tert*-leucine. (A) Effect of wild-type and variant FDHs on the reductive amination of TMP. (B) Effect of wild-type and variant FDHs on the reductive amination of TMP in the presence of 0.1 mM Cu^2+^. (C) The time course profile of residual activities in the biotransformation process in the presence of 0.1 mM Cu^2+^. All of the experiments were performed in triplicate. Standard deviations of the biological replicates are represented by error bars, and the error bars of some data are too small to be seen.

Asymmetric synthesis of l-*tert*-leucine, an important application example of FDH, was performed to evaluate the stability and catalytic efficiency of the variants that were used for coenzyme regeneration in this biotransformation. Residual activities of *Cbo*FDH, A10C_*fdh*_, I239C_*fdh*_, and A10C/I239C_*fdh*_ were 52.3%, 90.7%, 56.8%, and 92.5%, respectively, after 36 h of biotransformation. As shown in Fig. 3A, the biotransformation reached completion in 6 h with the variant A10C_*fdh*_ compared to 10 h for the wild-type *Cbo*FDH, which represented a 40% reduction in time. The reactions with the two other variants did not reach completion even after 12 h, but there were no significant differences in stability between the wild-type *Cbo*FDH and its variants after 24 h of biotransformation (data were not shown). The results indicated that the reduction of process time benefited from the increase in catalytic efficiency of variant A10C_*fdh*_ instead of the improvement of stability. However, in order to evaluate the potential contribution of variant enzymes to stability, the biotransformation of trimethyl pyruvate (TMP) to l-*tert*-leucine was performed in the presence of 0.1 mM Cu^2+^ that was used for promoting oxidation of sulfhydryl groups. As shown in Fig. 3B, the percent conversion followed a descending order of A10C_*fdh*_ (100.00%), A10C/I239C_*fdh*_ (63.68%), *Cbo*FDH (50.24%), and I239C_*fdh*_ (34.65%). The time course profile of residual activity is shown in Fig. 3C. Although it was under the condition of Cu^2+^-induced oxidation, the result indicated that to some extent, the variants A10C_*fdh*_ and A10C/I239C_*fdh*_ had good oxidation resistance in the biotransformation process compared to wild-type *Cbo*FDH and variant I239C_*fdh*_.

### Structure and molecular dynamics analysis.

Circular dichroism (CD) spectrum analysis was used to detect whether the introduction of disulfide bonds caused the secondary-structure changes of the enzyme *Cbo*FDH. The far-UV circular dichroism spectra (from 190 nm to 250 nm) of the wild-type enzyme and its derivatives displayed a series of similar curves (shown in Fig. S4). On-line analysis for protein circular dichroism spectra at the Dichroweb server showed that the secondary structures (helix, strand, turns, and unordered) have no obvious difference between the wild-type and variant enzymes.

We further analyzed whether there were changes of other factors besides disulfide bonds in the wild-type and variant enzymes with Discovery Studio (8). The result showed no variation of the amount and distribution of hydrogen bonds (803 pairs) and salt bridges (57 pairs), and the properties of variants under reducing conditions (the disulfide bonds were broken by the reducing reagent DTT) were similar to those of wild-type *Cbo*FDH (data not shown). These results further indicated that the disulfide bond plays a key role in the changes of variants' properties.

**FIG 4:**
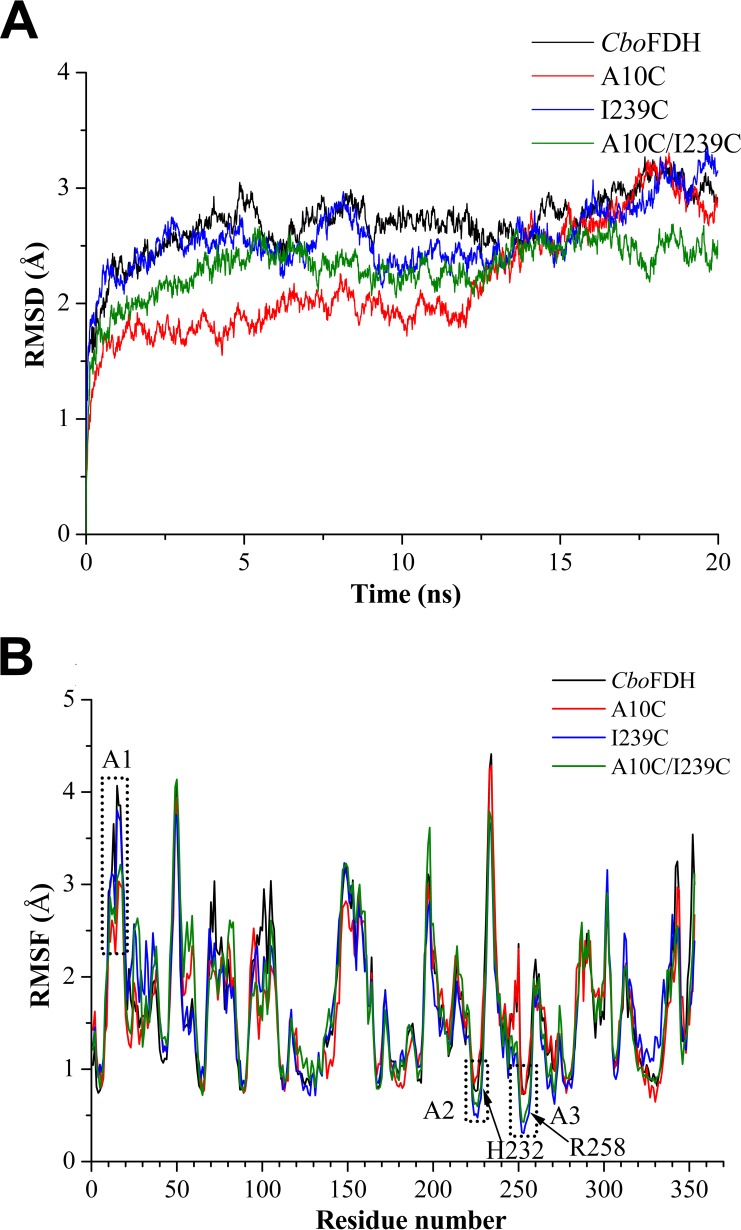
Molecular dynamics analysis of wild-type *Cbo*FDH and its variants. (A) RMSD values during 20 ns; (B) RMSF values calculated over the last 10 ns.

Molecular dynamics simulations of wild-type *Cbo*FDH and its derivatives at 333 K were carried out for 20 ns. As shown by the root mean square deviation (RMSD) calculated for the Cα atoms (Fig. 4A), all enzymes displayed an equilibrium state within the last 12.5 ns. Generally, lowering the overall RMSD is one way to modify the enzyme structure to make it more rigid and consequently enhance its stability (19, 20). The variants A10C_*fdh*_ and A10C/I239C_*fdh*_ had lower RMSD values than wild-type *Cbo*FDH, suggesting that their structures were more stable. Taking the root mean square fluctuation (RMSF) value as the index (Fig. 4B), the hyperthermosensitive region, referred to as region A1 (residues A10 to E18), was more stable in the variants A10C_*fdh*_ and A10C/I239C_*fdh*_ than in the wild-type *Cbo*FDH and variant I239C_*fdh*_. Thus, the crucial role of the disulfide bond A10C-C23 in stability was also supported by the molecular dynamics simulation results. The relatively stable regions in the NAD^+^ binding domains, referred to as regions A2 (residues D223 to H232) and A3 (residues G250 to R258), were more stable in the variants I239C_*fdh*_ and A10C/I239C_*fdh*_ than in the wild-type *Cbo*FDH and variant A10C_*fdh*_.

## DISCUSSION

*Cbo*FDH is a biocatalyst for regeneration of NADH, is used for industrial production of l-*tert*-leucine (21), and has been extensively studied to be applied in the production of other chiral compounds (22–24). Native FDH has been modified by some approaches, such as increasing the stability (25–27) and improving kinetic properties (28, 29), to improve its application performance. In this study, we first employed disulfide bonds to relieve the oxidation of the free sulfhydryl groups of *Cbo*FDH and developed an excellent variant, A10C_*fdh*_, that showed a significant increase in copper ion and acid resistance along with an increase of thermal stability and specific activity. Previous reports (4, 30) and an accurate structural model of *Cbo*FDH (31, 32) provide more direct and readily available information related to enzyme properties for site-directed mutagenesis. Computational design methods provide more precise guidance for the engineering of disulfide bonds (15, 16) and make mutations more efficient. According to the results of SDS-PAGE (see Fig. S2 in the supplemental material), the determination of free cysteines (Table 1), and copper ion resistance assays (Fig. 1), we confirmed that disulfide bonds of A10C-C23 and I239C-C262 were successfully introduced into derivatives of *Cbo*FDH. The improvement of Cu^2+^ tolerance also indicated that the oxidation of the free cysteine was removed due to construction of disulfide bonds between the sulfhydryl groups in variant enzymes.

**FIG 5:**
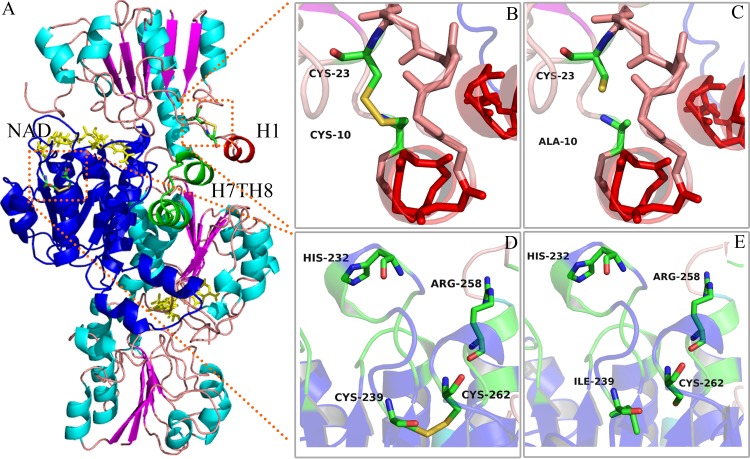
Model analysis of the wild-type *Cbo*FDH and variant enzymes. (A) Model of A10C/I239C_*fdh*_ containing disulfide bonds A10C-C23 and I239C-C262. The Rossmann fold motif is depicted in blue. (B and C) Local comparison of A10C_*fdh*_ and *Cbo*FDH. (D and E) Local comparison of I239C_*fdh*_ and *Cbo*FDH.

To understand the factors contributing to the improved stability of variant enzymes, we evaluated the secondary structures and other intramolecular interactions with CD and Discovery Studio. A previous report indicated that the improved stability of enzymes is based on minor structural changes (33). Our results showed that the introduction of the disulfide bonds did not bring obvious changes in the secondary structures or other intramolecular interactions of the enzyme. According to the results of enzymatic copper ion resistance (Fig. 1), thermal stability (Fig. 2), and pH stability (Fig. S3) assays, we concluded that C23 was paired with the mutation residue C10 to form the disulfide bond A10C-C23, which made a greater contribution to the improvement of enzyme stability than I239C-C262. It is reported that the thiol group of C23 is directly exposed to the solvent and the thiol group of C262 is buried inside the protein (Fig. 5C and E), so the free C23 is more susceptible to oxidation than C262 (4, 32). In our work, the construction of a disulfide bond between A10C and C23 not only solved the problem caused by unpaired C23 oxidation but also improved the stability of *Cbo*FDH.

To explore the reasons for changes in specific activity and catalytic efficiency of *Cbo*FDH, we analyzed the positions of disulfide bonds A10C-C23 and I239C-C262 in *Cbo*FDH. As shown in Fig. 5A, amino acid residues A10 and C23 were located on both sides of an α-helix (residues K12 to D16, termed H1 and belonging to the hyperthermosensitive region A1), while H1 plays a crucial role in formation of *Cbo*FDH dimers and the closure of the active-site channel with a helix-turn-helix motif (residues F138 to K157; termed H7TH8) (32). The closure of the active-site channel is crucial for catalysis, as it can avoid the interference of hydride transfer reaction by surrounding solvent water molecules (34). The formation of the A10C-C23 disulfide bond strengthened the placement of helix H1 (Fig. 5B), which might make the interaction between helix H1 and motif H7TH8 more stable. On the other hand, this effect was also reflected in the improvement of stability and acid resistance of the variants A10C_*fdh*_ and A10C/I239C_*fdh*_. The stability of dimerization caused the end of the active-site channel to be locked in very tight packing, and the catalytic reaction of enzymes might be carried out more smoothly. This was reflected in the experimental results that the specific activity and catalytic efficiency of variant enzyme A10C_*fdh*_ increased by 32% and 33%, respectively, although the NAD^+^ affinity declined slightly. Amino acid residues I239 and C262 were located in the random coil of the Rossmann fold motif (35) (Fig. 5A). As shown in Fig. 5D, amino acid residue H232, which interacted with the adenine ring of NAD^+^, was on top of I239, the formation of disulfide bond I239C-C262 reduced the flexibility of the NAD^+^ binding pocket (Fig. 4B), and the NAD^+^ affinity of the variant enzymes I239C_*fdh*_ and A10C/I239C_*fdh*_ had declined. Amino acid residue R258, which binds formate in the active site, was on top of C262, the formation of disulfide bond I239C-C262 made the position of R258 more fixed (Fig. 4B), and the formate affinity of the variant enzymes I239C_*fdh*_ and A10C/I239C_*fdh*_ was improved. Through the comprehensive analysis described above, we could conclude that the disulfide bond A10C-C23 played a positive role in increasing the catalytic efficiency and stability of *Cbo*FDH, whereas the disulfide bond I239C-C262 made a negative contribution by reducing the NAD^+^ affinity and catalytic efficiency of *Cbo*FDH.

In conclusion, the catalytic efficiency and stability of NAD^+^-dependent formate dehydrogenase from *Cbo*FDH were remarkably improved via the rational construction of the disulfide bonds. The reduction of l-*tert*-leucine synthesis process time (Fig. 3A) benefited from the increase in catalytic efficiency of variant A10C_*fdh*_ instead of the improvement of stability. Stable variant FDH can also improve its application performance, such as FDH storage, or the biotransformation system involving by-product oxygen or Cu^2+^-activated enzyme. (These hypotheses are subject to confirmation.) The superiority on activity and stability of variant A10C_*fdh*_ in a complex environment (such as the presence of Cu^2+^) will make it more reliable in industrial application. Our strategy is also expected to be applicable to the engineering of other industrial enzymes to avoid inactivation caused by the oxidation of free cysteine and improving their performance.

## MATERIALS AND METHODS

### Strains, plasmids, and materials.

The sequence of the NAD^+^-dependent formate dehydrogenase gene (*fdh*) from Candida boidinii was obtained from GenBank (GI 152207662 [DQ458777.2]) and synthesized by Sangon (Shanghai, China). Plasmid pET-28a(+) (Novagen) was used as the expression vector for the *Cbo*FDH expression and mutagenesis studies. E. coli JM109 was used as the host for gene cloning, and E. coli BL21(DE3) was used as the host for the expression of *Cbo*FDH and its variants. PrimeSTAR HS DNA polymerase, restriction enzymes, and T4 DNA ligase were purchased from TaKaRa (Dalian, China). An agarose gel DNA recovery kit, plasmid extraction kit, and Bradford protein assay kit were purchased from Generay (Shanghai, China). All other reagents of analytical grade or higher quality were obtained from commercial sources.

### Site-directed mutagenesis.

Site-directed mutagenesis of the *fdh* gene was performed using the overlap extension PCR method (36). The primers used for site-directed mutagenesis are listed in Table 3. Final PCR products were ligated into the plasmid pET-28a(+) and sequenced. The recombinant plasmids were transformed into E. coli BL21(DE3) for expression.

**TABLE 3:** Primers used in this study

Primer	Sequence^*a*^ (5′→3′)
*fdh*-F	GGAATTCATGAAGATCGTTTTAGTCTTATAC (EcoRI)
*fdh*-R(A10C-R)	CCCTCGAGTTATTTCTTATCGTGCTTACCATAA (XhoI)
A10C-F	GGAATTCATGAAGATCGTTTTAGTCTTATACGAT***TGT***GGTAAACAC (EcoRI)
I239C-F	GGTACAAAAGGTTTA***TGT***AACAAGGAATTATTG
I239C-R	CAATAATTCCTTGTT***ACA***TAAACCTTTTGTACC

a Mutant sites are shown in boldface and italics; restriction sites are underlined (restriction enzymes are in parentheses).

### Protein expression and purification.

The recombinant strains were initially cultured in lysis broth (LB) medium containing 50 μg/ml kanamycin at 37°C overnight as seed liquid and then inoculated into TY medium (37) containing the same amount of kanamycin with a 1% inoculation. Cultures were grown at 37°C until the optical density at 600 nm reached about 0.6. Isopropyl β-d-1-thiogalactopyranoside was added to a final concentration of 0.5 mM to induce the expression of the target protein at 25°C for 16 h. The cells were harvested by centrifuging at 8,000 × *g*, 4°C, for 10 min and then were washed twice with 50 mM phosphate-buffered saline (PBS) buffer (pH 7.5). The harvested cells were sonicated and centrifuged at 10,000 × *g* for 30 min at 4°C to remove the cell debris. The supernatant was used for enzyme activity assays and purification.

The wild-type *Cbo*FDH and its variants were subjected to metal affinity chromatography on a 1-ml HisTrap FF column (GE Life Sciences, USA), which was performed on an AKTA purifier system. A linear gradient of 0 to 500 mM imidazole in buffer A was used to elute the adsorbed proteins. The fractions showing FDH activity were pooled for SDS-PAGE analysis. The protein concentration was determined with a Bradford protein assay kit.

### Verification of disulfide bond formation.

SDS-PAGE (under reducing and nonreducing conditions as described previously [38]) was used to determine whether disulfide bonds were present in the variant proteins by comparing the mobility of enzyme samples under reducing conditions to that under nonreducing conditions. Samples (16 μl) were pretreated via mixing with 4 μl of 5× SDS-PAGE loading buffer in a 0.25-ml centrifuge tube in the presence (reducing conditions) or absence (nonreducing conditions) of 100 mM 1,4-dithiothreitol (DTT).

To further confirm the presence of disulfide bonds in variant proteins, the amount of free cysteines in proteins was determined by assaying free thiol content using dithionitrobenzoic acid (DTNB) (39). Protein samples (1 ml) were mixed with 5 ml DTNB reagent (0.1 mM DTNB solution, 0.5 mM Na_2_HPO_4_ buffer [pH 7.0], and 0.25 M Tris-HCl buffer [pH 8.3]), and the mixtures were incubated for 10 min at room temperature. After incubation, the absorbance at 412 nm was recorded against a blank. A molar extinction coefficient of 13,600 M^−1^ cm^−1^ was used for conversion of absorbance to thiol concentration. The sample blank and reagent blank were prepared to correct for the color from protein and reagent solution. The total number of free cysteines (in one protein molecule) was calculated using the following equation: *X* · Mr_CYS_/Mr_FDH_ ≈ *C*_CYS_ · Mr_CYS_, where *X* is the total number of free cysteines (in one protein molecule), Mr_CYS_ is the relative molecular mass of cysteine, Mr_FDH_ is the relative molecular mass of wild-type FDH or variants, and *C*_CYS_ is the concentration of free cysteines (millimoles per microgram of protein).

### Enzyme activity and copper ion resistance assays.

FDH activity was determined using the method described by Slusarczyk (4). The assay mixture contained 167 mM sodium formate and 1.67 mM NAD^+^ in 100 mM potassium phosphate buffer, pH 7.0, and the reaction was started by addition of limiting amounts of FDH. The increase in absorbance at 340 nm was recorded and activities were calculated as enzyme units (i.e., micromoles per minute) by using ε = 6,220 M^−1^ · cm^−1^ for NADH at 340 nm.

Copper ion resistance assays of the purified enzyme were carried out in the presence of different concentrations of copper chloride (0, 1, 2.5, 5, 7.5, 10, 12.5, and 15 mM) in 100 mM Tris-HCl buffer, pH 7.0. The potassium phosphate buffer of the assay mixture mentioned above was replaced with Tris-HCl (100 mM, pH 7.0) buffer to prevent cupric phosphate precipitation. Activities were measured at pH 7.0 and 30°C. All of the assays were performed in triplicate.

### Determination of kinetic parameters.

The kinetic constants (*K_m_*, *k*_cat_, and *k*_cat_/*K_m_*) were measured under conditions where only one substrate (formate or NAD^+^) was limited (31). *K_m_* for NAD^+^ was measured by variation of the NAD^+^ concentration from 0.02 to 12 mM at a formate concentration of 200 mM, and the *K_m_* for formate was determined by variation of the formate concentration from 1 to 215 mM at an NAD^+^ concentration of 10 mM. The corresponding data were fit to the Michaelis-Menten equation to obtain the kinetic constants *k*_cat_ and *K_m_* for both substrates. All kinetic constants were the mean values derived from triplicate measurements.

### Measurement of thermal and pH stability.

Thermal stability of the wild-type and variant enzymes was monitored after incubation in Tris-HCl buffer (50 mM, pH 7.0) for 20 min at a range of temperatures from 30°C to 70°C (Tris-HCl buffers were prepared at the temperature at which they were used). Residual enzyme activities then were determined in potassium phosphate buffer (100 mM, pH 7.0) at 30°C. The *T*_50_ value was the half-inactivation temperature at which 50% of the initial enzyme activity was lost after heat treatment. The *T*_50_ values were evaluated from the plots of residual relative activity (percent) versus temperature (degrees Celsius) (40). Purified enzymes were incubated in Tris-HCl buffer (50 mM, pH 7.0) at 60°C for different times to detect the half-lives of thermal inactivation (*t*_1/2_) using a PCR instrument to control temperature and time accurately. The first-order rate constant, *k*, was obtained from the slope of a semilogarithmic plot of incubation time versus residual activity (see Fig. S1 in the supplemental material), and the values of *t*_1/2_ at 60°C were calculated using the equation *t*_1/2_ = ln2/*k* (4).

To determine the pH stability of wild-type *Cbo*FDH and its variants, the following buffer systems were prepared: 50 mM citrate-sodium citrate buffer (pH 3.0 to 6.0), 50 mM phosphate-buffered saline (PBS) buffer (pH 6.0 to 8.0), 50 mM Tris-HCl (pH 8.0 to 10.0), 50 mM glycine-NaOH (pH 10.0 to 11.0), and 50 mM Na_2_HPO_4_-NaOH (pH 11.0 to 12.0). The purified enzyme was incubated in the above-described buffers at 37°C for 1 h, and residual activity was measured in potassium phosphate buffer (100 mM, pH 7.0) at 30°C. All assays were performed in triplicate.

### Asymmetric synthesis with coenzyme regeneration.

The enzymatic reaction system for l-*tert*-leucine synthesis contained 0.3 M trimethyl pyruvate (TMP), 0.6 M ammonium formate, 0.3 mM NAD^+^, 6.0 U/ml leucine dehydrogenase, and 1.5 mg/ml formate dehydrogenase (wild-type *Cbo*FDH or its variants) in 0.1 M Tris-HCl buffer, pH 8.5, and reactions were performed at 30°C at 200 rpm in the presence or absence of 0.1 mM Cu^2+^. In order to efficiently evaluate the stability of variants, Cu^2+^ was used to promote oxidation of cysteine residues. The determination of substrate and l-*tert*-leucine levels was performed on an Agilent LC1260 high-performance liquid chromatography (HPLC) system as described by Liu et al. (41). All assays were performed in triplicate.

### CD for protein structure analysis.

Circular dichroism (CD) analysis of wild-type *Cbo*FDH and its variants was performed on a MOS-450/AF-CD-STP-A spectrometer (Bio-Logic, France). The proteins were diluted to 100 μg/ml (2.30 μM) in ultrapure water. Spectra were recorded from 190 nm to 250 nm with a 1-mm cell and a bandwidth of 1 nm over three scans at a scan rate of 120 nm/min. The results were analyzed on the Dichroweb server (http://dichroweb.cryst.bbk.ac.uk/html/process.shtml).

### Structure and molecular dynamics analysis.

The structural model of *Cbo*FDH (5dn9) was obtained from the RCSB Protein Data Bank (PDB). The Disulfide by Design (15) platform was applied to predict the residues for possible formation of disulfide bonds with the original cysteine residues in protein based on an accurate model of wild-type *Cbo*FDH. Intramolecular interactions (disulfide bonds, hydrogen bonds, and salt bridges) in wild-type *Cbo*FDH and its variants were counted by Discovery Studio 2.5 (Accel-rys, San Diego, CA, USA). The program PYMOL was used for model viewing and image processing.

Molecular dynamics simulation was conducted to analyze the thermal fluctuation of *Cbo*FDH and its derivatives at 333 K by using YASARA software (http://www.yasara.org) with an Amber99 force field. The cutoff distance, 7.9 Å, of the long-range electrostatics of particle mesh Ewald (PME) and Van der Waals interactions were employed. Trajectory analyses of data were performed with YASARA.

## Supplementary Material

Supplemental material
