# Supplementary material for: Elimination of a Free Cysteine by Creation of a Disulfide Bond Increases the Activity and Stability of Candida boidinii Formate Dehydrogenase
Source: Appl Environ Microbiol. 2016 Dec 30;83(2):e02624-16. doi: 10.1128/AEM.02624-16 (PMC5203636; doi:10.1128/AEM.02624-16)
Supplement: Supplemental material [file supp_83_2_e02624-16__index.html]

Elimination of a Free Cysteine by Creation of a Disulfide Bond Increases the Activity and Stability of Candida boidinii Formate Dehydrogenase — Supplemental material 

# Elimination of a Free Cysteine by Creation of a Disulfide Bond Increases the Activity and Stability of Candida boidinii Formate Dehydrogenase

## Supplemental material

- Supplemental file 1 -

  The exponential fitting curves of the data points of stability analysis (Fig. S1), SDS-PAGE analysis of purified wild-type and mutant enzymes under reducing and nonreducing conditions (Fig. S2), enzyme inactivation assay at different pHs for 1 h (Fig. S3), and far-UV CD spectra of wild-type *Cbo*FDH and its variants (Fig. S4).

  PDF, 330K
