# Supplementary material for: Elimination of a Free Cysteine by Creation of a Disulfide Bond Increases the Activity and Stability of Candida boidinii Formate Dehydrogenase
Source: Appl Environ Microbiol. 2016 Dec 30;83(2):e02624-16. doi: 10.1128/AEM.02624-16 (PMC5203636; doi:10.1128/AEM.02624-16)
Supplement: Supplemental material [file AEM.02624-16_zam999117620s1.pdf]

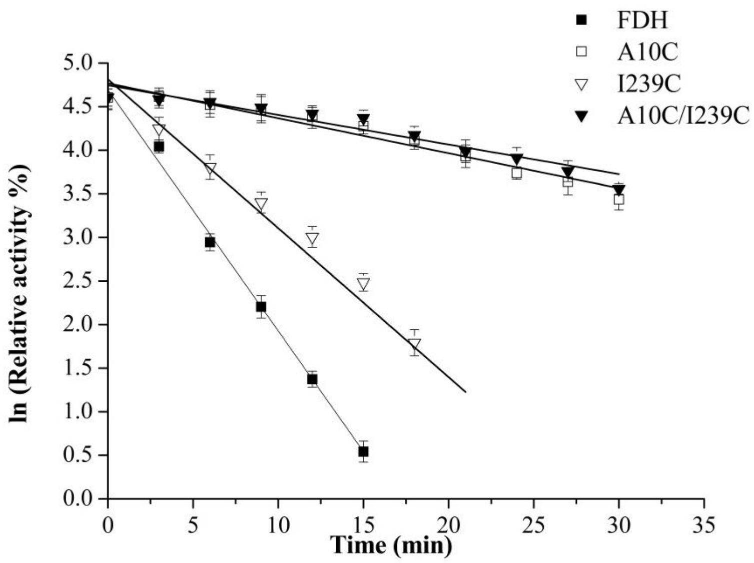

Fig. S1 The exponential fitting curves of the data points of stability analysis. Thermal stability analysis of wild-type and variants was performed at 60 ℃, pH 7.0. (Standard deviations of the biological replicates were represented by error bars.)

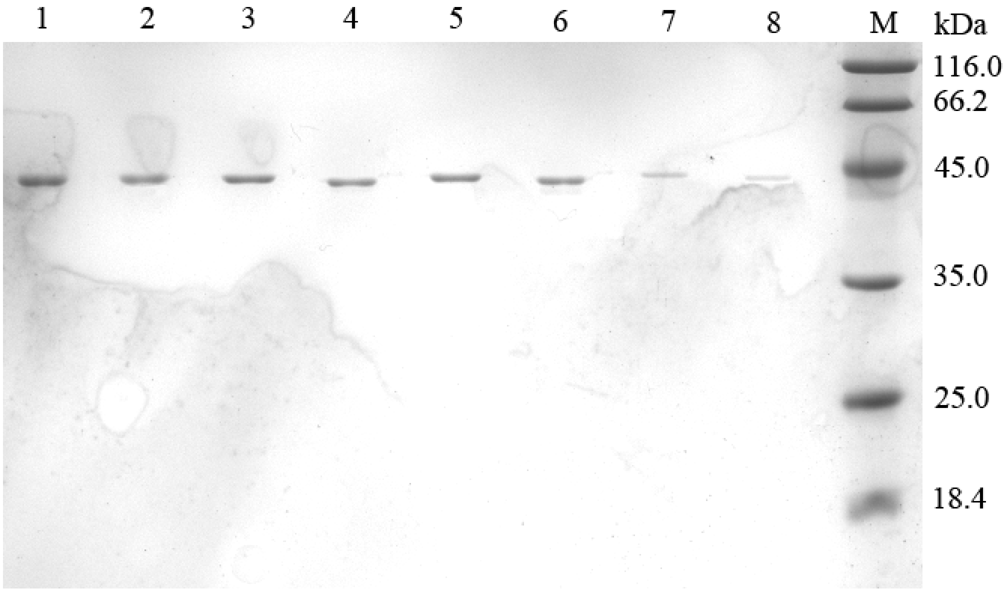

Fig. S2 SDS-PAGE analysis of purified wild-type and mutant enzymes under reducing (lanes: 1, 3, 5 and 7) and non-reducing (lanes: 2, 4, 6 and 8) conditions. Lanes: 1 and 2 the wild-type *Cbo*FDH; 3 and 4 A10C$_{fdh}$; 5 and 6 I239C$_{fdh}$; 7 and 8 I239C/A10C$_{fdh}$; M, molecular mass marker.

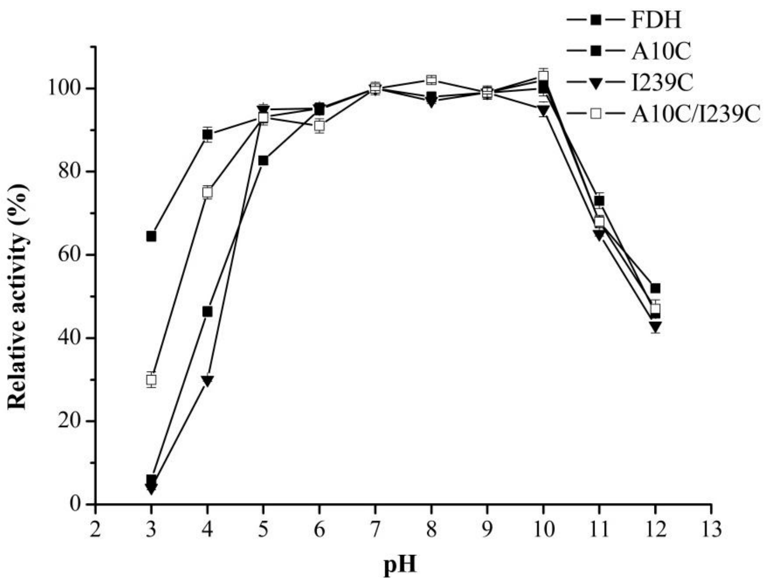

Fig. S3 Enzyme inactivation assay at different pH for 1 h. (Standard deviations of the biological replicates were represented by error bars.)

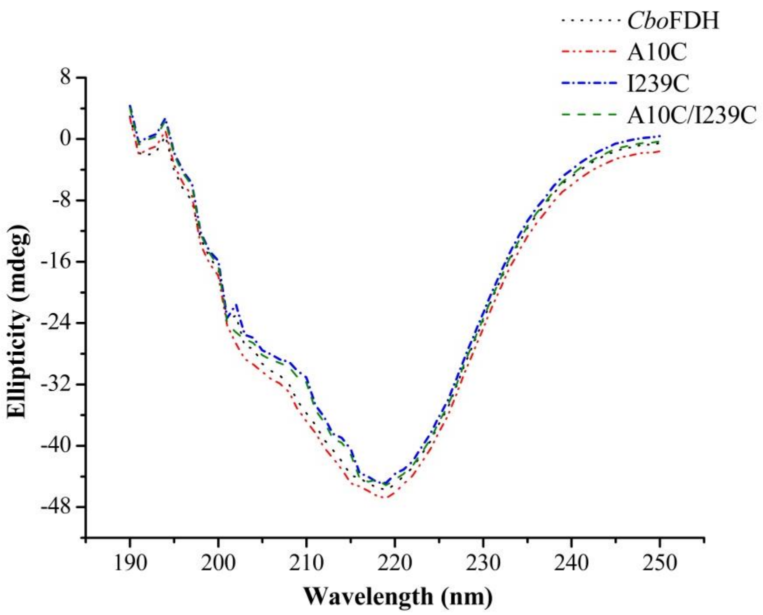

Fig. S4 Far-UV CD spectra of wild-type *Cbo*FDH and its variants.
